# Supplementary material for: Deep learning for universal linear embeddings of nonlinear dynamics
Source: Nat Commun. 2018 Nov 23;9:4950. doi: 10.1038/s41467-018-07210-0 (PMC6251871; doi:10.1038/s41467-018-07210-0)
Supplement: Supplementary file 1 — Supplementary Information [file 41467_2018_7210_MOESM1_ESM.pdf]

Deep learning for universal linear embeddings of nonlinear dynamics

Lusch, et al.

Supplementary Note 1 – Additional details for results

Discrete spectrum example

Supplementary Figure 1 shows the average prediction error versus the number of prediction steps. Even for a large number of steps, the error is quite small, giving good prediction. This figure also demonstrates prediction performance on example trajectories. The eigenfunctions for this example are shown in Supplementary Figure 2. We see that one is quadratic and the other is linear. This is expected because we can analytically derive that $y_1 = x_1$ and $y_2 = x_2 - bx_1^2$ is a pair of eigenfunctions for this system, where $b = \frac{-\lambda}{2\mu - \lambda}$. When the eigenvalues are allowed to vary with the auxiliary network used for continuous spectrum systems, the eigenvalues remain relatively constant, near the true values of -0.05 and -1, as shown in Supplementary Figure 3.

Nonlinear pendulum

The nonlinear pendulum is one of the simplest examples that exhibits a continuous eigenvalue spectrum. Using the auxiliary network, we allow the frequency ω of the Koopman eigenvalues to vary continuously with the embedded coordinates y_1 and y_2, as shown in Supplementary Figure 4. The frequency ω varies smoothly with the radius $\sqrt{y_1^2 + y_2^2}$, from around -0.95 to -0.4 as the energy is increased. When the damping rate is also allowed to vary continuously, it remains nearly constant around the value of $\mu = 0$, since the system is conservative.

Fluid flow on attractor

For the final example, we consider the nonlinear fluid vortex shedding behind a cylinder. We begin by considering dynamics on the attracting manifold. When we train the network with trajectories on the slow manifold, we are able to identify a single conjugate eigenfunction pair, shown in Supplementary Figure 6.

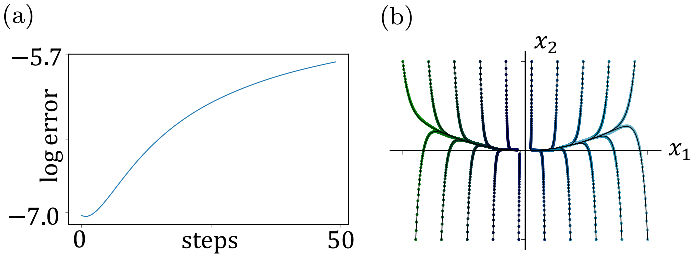

Supplementary Figure 1: (a) The average $\log_{10}$ prediction error as the number of prediction steps increases for the discrete spectrum example. (b) For each trajectory, we show how many steps the network can take before reaching 10% relative error.

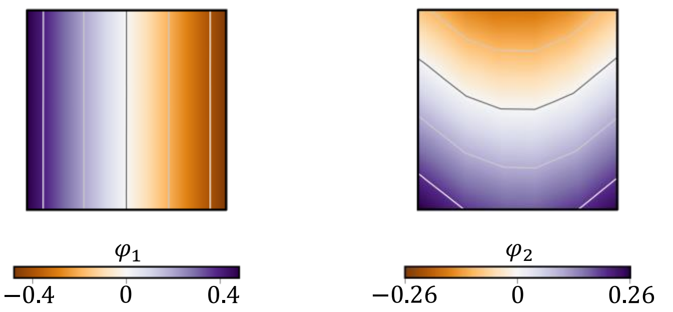

Supplementary Figure 2: Eigenfunctions for discrete spectrum example.

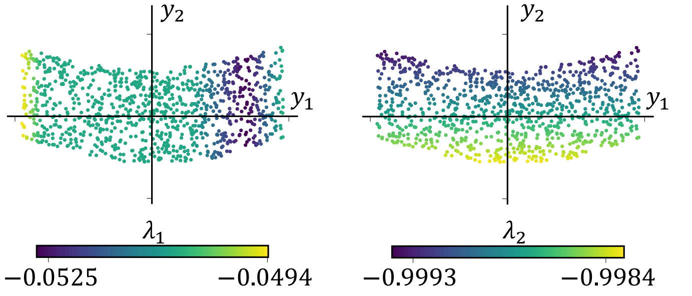

Supplementary Figure 3: When the eigenvalues of the discrete spectrum example are allowed to vary in terms of y_1 and y_2, they remain relatively constant; i.e., they are close to the true values $-.05$ and -1 as expected.

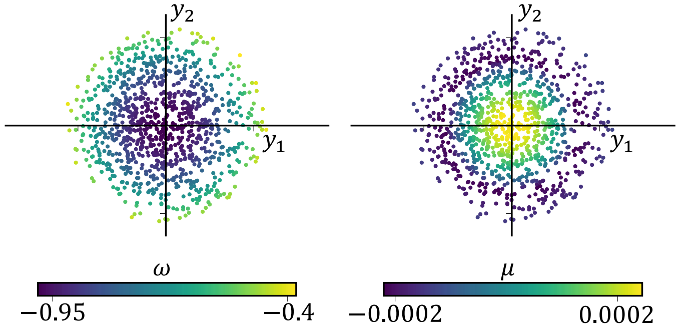

Supplementary Figure 4: Eigenvalues for the pendulum vary in terms of y_1 and y_2. Note that the frequency decreases as the radius increases, and $\mu \approx 0$.

Fluid flow off attractor

We now consider the case where we train a network using trajectories that start off of the attracting slow manifold. Supplementary Figure 7 shows the average prediction error versus the number of steps. Although the loss function only penalized 30 prediction steps in the future ($S_p = 30$), the error remains small for all 100 steps. The figure also shows the embedding of a trajectory in y coordinates. Although this network's training data includes data off the attractor, this network's embedding is similar to the embedding from the previous case. (See Figure 5 in the main paper.)

The eigenfunctions are shown in Supplementary Figure 8, where it can be seen that the mode shapes match those in the on-attractor data in Supplementary Figure 6.

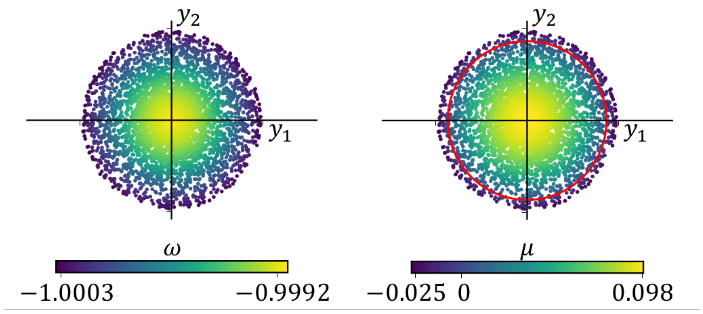

Supplementary Figure 5: Continuous eigenvalues as a function of y_1 and y_2. Note that the frequency $\omega \approx -1$. The parameter μ shows growth inside the limit cycle (marked in red) and decay outside the limit cycle.

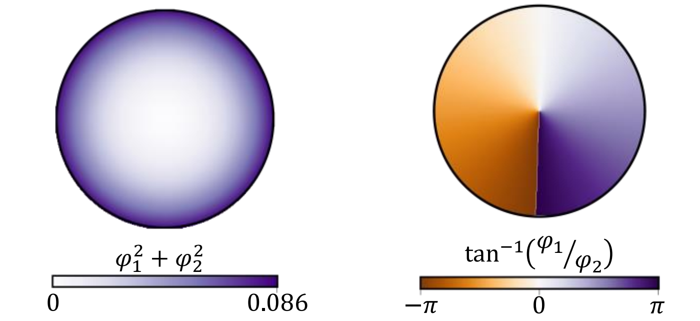

Supplementary Figure 6: Magnitude and phase of the eigenfunctions for the fluid flow on the attracting slow manifold.

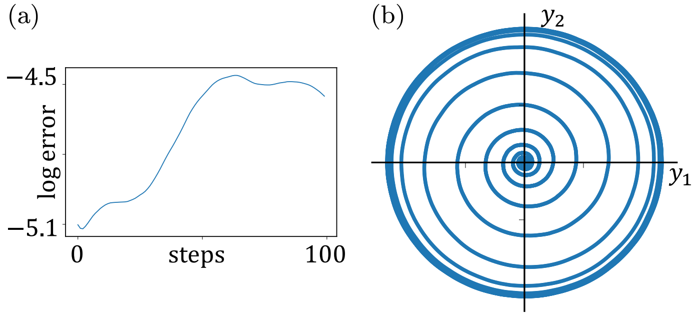

Supplementary Figure 7: (a) The average $\log_{10}$ prediction error as the number of prediction steps increases for the fluid flow example with trajectories starting off the attractor. (b) A trajectory on the attractor in linear coordinates y_1 and y_2.

The continuously varying eigenvalues are shown in Supplementary Figure 9. Again, similar to the on-attractor case, the damping μ varies considerably with radius, while the frequency is very nearly a constant -1.

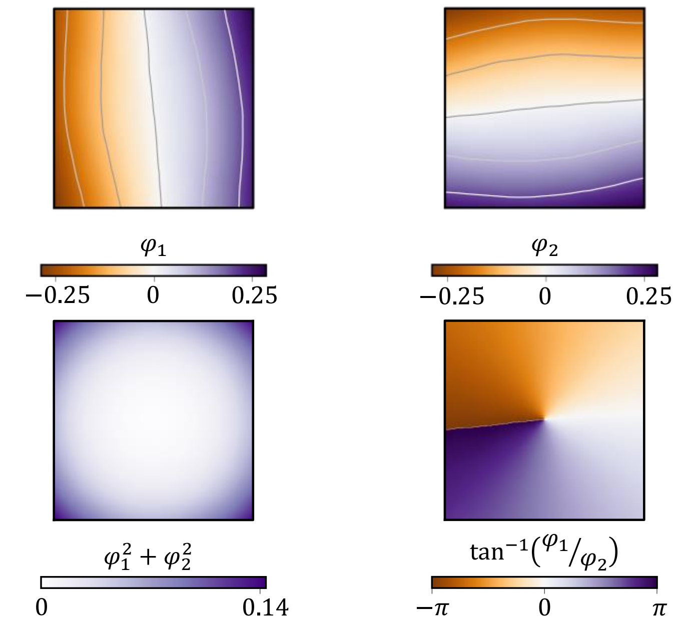

Supplementary Figure 8: Eigenfunctions for the fluid flow example for trajectories starting off the attractor, corresponding to the complex conjugate pair of eigenvalues; the second row contains the magnitude and phase of those eigenfunctions.

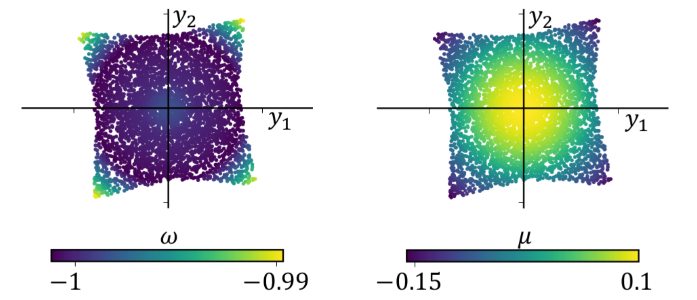

Supplementary Figure 9: Parameter variations for the complex eigenvalues in terms of y_1 and y_2. Note that this is a natural extension of Supplementary Figure 5, which is limited to data on the bowl.
